# Gut microbiota-mediated lysophosphatidylcholine generation promotes colitis in intestinal epithelium-specific *Fut2* deficiency

**DOI:** 10.1186/s12929-021-00711-z

**Published:** 2021-03-15

**Authors:** Xuelian Tang, Weijun Wang, Gaichao Hong, Caihan Duan, Siran Zhu, Yuen Tian, Chaoqun Han, Wei Qian, Rong Lin, Xiaohua Hou

**Affiliations:** Department of Gastroenterology, Union Hospital, Tongji Medical College, Huazhong University of Science and Technology, Wuhan, 430022 China

**Keywords:** *Fut2*, IBD, Gut microbiota, LPC

## Abstract

**Background and aims:**

Previous study disclosed *Fucosyltransferase* 2 (*Fut2*) gene as a IBD risk locus. This study aimed to explore the mechanism of *Fut2* in IBD susceptibility and to propose a new strategy for the treatment of IBD.

**Methods:**

Intestinal epithelium-specific *Fut2* knockout (*Fut2*^△IEC^) mice was used. Colitis was induced by dextran sulfate sodium (DSS). The composition and diversity of gut microbiota were assessed via 16S rRNA analysis and the metabolomic findings was obtained from mice feces via metabolite profiling. The fecal microbiota transplantation (FMT) experiment was performed to confirm the association of gut microbiota and LPC. WT mice were treated with Lysophosphatidylcholine (LPC) to verify its impact on colitis.

**Results:**

The expression of *Fut*2 and α-1,2-fucosylation in colonic tissues were decreased in patients with UC (UC vs. control, *P* = 0.036) and CD (CD vs. control, *P* = 0.031). When treated with DSS, in comparison to WT mice, more severe intestinal inflammation and destructive barrier functions in *Fut2*^△IEC^ mice was noted. Lower gut microbiota diversity was observed in *Fut2*^△IEC^ mice compared with WT mice (*p* < 0.001). When exposed to DSS, gut bacterial diversity and composition altered obviously in *Fut2*^△IEC^ mice and the fecal concentration of LPC was increased. FMT experiment revealed that mice received the fecal microbiota from *Fut2*^△IEC^ mice exhibited more severe colitis and higher fecal LPC concentration. Correlation analysis showed that the concentration of LPC was positively correlated with four bacteria—*Escherichia*, *Bilophila*, *Enterorhabdus* and *Gordonibacter*. Furthermore, LPC was proved to promote the release of pro-inflammatory cytokines and damage epithelial barrier in vitro and in vivo*.*

**Conclusion:**

*Fut*2 and α-1,2-fucosylation in colon were decreased not only in CD but also in UC patients. Gut microbiota in *Fut2*^△IEC^ mice is altered structurally and functionally, promoting generation of LPC which was proved to promote inflammation and damage epithelial barrier.

**Supplementary Information:**

The online version contains supplementary material available at 10.1186/s12929-021-00711-z.

## Introduction

Inflammatory bowel disease (IBD) is a chronic inflammatory gastrointestinal disorder and a global public health concern [1, 2]. It is widely accepted that the etiology of IBD depends on dysregulation of immune responses interacting with gut microbiota and environmental factors in genetically susceptible individuals and remains to be further explored [3, 4].

In recent years, genome wide association study (GWAS) studies have advanced our knowledge of the heritability of IBD and the list of risk genes is rapidly expanding [5, 6]. ATG16L1, IL23R, PRDM1 and CARD9 genes, etc., are discovered to be associated with IBD [7]. It has been newly reported that *fucosyltransferase 2* (*Fut2*) non-secretor of state (individuals lacking a functional copy of *Fut2* are known as non-secretors) is associated with Crohn's Disease (CD) susceptibility [5, 6, 8], however the mechanism remains unknown.

*Fut2* is one of the enzymes responsible for the addition of fucose to proteins or lipids by α-1,2-fucosylation on the intestinal mucosa, which can act as both an attachment site and carbon source for intestinal bacteria [9–11]. Tong et al. [12] analyzed the gut microbiota in healthy subjects and they found that the gut microbiota was altered in non-secretor. Interestingly, previous research revealed that in patients with primary sclerosing cholangitis, *Fut2* non-secretors exhibited a different composition of bile microbiota compared to *Fut2* secretors [13]. Furthermore, Guntram et al. [14] demonstrated that *Salmonella* expresses Std fimbriae in the gastrointestinal tract. They also demonstrated that *Salmonella*-triggered intestinal inflammation and colonization are dependent on Std-fucose interaction. These findings suggests potential regulatory effect of *Fut2* on gut microbiota. But, few has been studied in the changes of gut microbiota in the state of IBD or experimental colitis.

It is known that gut microbiota plays vital biological roles in healthy hosts, including maintenance of immune homeostasis, modulation of intestinal development and enhanced metabolic capabilities [15–18]. Besides, metabolomics studies have revealed large effects of the gut microbiota on host metabolism [19, 20]. Therefore, besides the direct effect, gut microbiota may play an indirect effect on intestinal injury by changing metabolites. Tong M et al. [12] revealed the disturbance of energy metabolism in the microbiome of non-secretors individuals, including lipid and carbohydrate metabolism, cofactor and vitamin metabolism and glycan biosynthesis. However, the correlation between the changes in metabolites and the IBD susceptibility in non-secretor has not been studied. So we designed the study to investigate the gut microbiota and the metabolism in WT and *Fut2*^△IEC^ mice in the presence or absence of DSS, in order to figure out the mechanism of *Fut2* deficiency in IBD susceptibility.

In this study, intestinal epithelium-specific *Fut2* knockout mice (*Fut2*^△IEC^ mice) were constructed to evaluate underlying mechanisms of *Fut2* in IBD. The gut microbiota and metabolism in WT and *Fut2*^△IEC^ mice with/without DSS-induced colitis were studied. The fecal microbiota transplantation (FMT) experiment was performed to confirm the association of gut microbiota and the metabolism. Collectively, our data revealed that intestinal epithelium-specific *Fut2* deficiency mice were susceptible to colitis through modulation of gut microbiota and generation of LPC.

## Methods

### Patient selection

The colon tissues of healthy subjects (n = 8) and patients diagnosed with CD (n = 5) or UC (n = 16) were collected. The patients met the following criteria: CDAI score ≥ 150 for CD patients, while Mayo score ≥ 3 for UC patients; if colonoscopy did not reveal any abnormalities, subjects were included in the healthy group. The study was approved by the Ethics Committee of Tongji Medical College, Huazhong University of Science and Technology. All patients provided written informed consent for and their information had been anonymized and de-identified.

### Animal experiment

#### Mice

We used Pvillin-Cre recombinase transgenic C57BL/6 mice (Pvillin-Cre TG mice) and *Fut2*^flox/flox^ C57BL/6 mice (purchased from GemPharmatech Co. Ltd) to cross and generate mice with *Fut2* gene specifically deleted in intestinal epithelial cell (Pvillin-Cre + *Fut2*^flox/flox^ mice, abbreviated as *Fut2*^△IEC^). The data validates the knock-out of *Fut2* in intestinal epithelial cells along with the result in loss of α-1,2-fucosylation in the tissue were shown in Additional file 1: Figure S1. *Fut2*^△IEC^ male mice (8–10 weeks old) were used for further experiments. Wild-type C57BL/6 male mice were purchased from Beijing Vital River Laboratory Animal Technology Co., Ltd., Beijing, China and fed for acclimatization. All mice were housed in the specific pathogen free (SPF) grade facility of Huazhong University of Science and Technology and maintained at 12 h light/dark cycles with free access to food and water. All animal studies were approved by the Animal Experimentation Ethics Committee of Huazhong University of Science and Technology and performed in accordance with national and EU guidelines.

### Model establishment

WT mice and *Fut2*^△IEC^ mice were randomly divided into four groups—WT control, WT DSS, *Fut2*^△IEC^ control *and Fut2*^△IEC^ DSS. Mice in DSS groups (WT DSS and *Fut2*^△IEC^ DSS), colitis was induced by 3% (w/v) dextran sulfate sodium (DSS, 36–50 kDa; MP Biomedicals, Santa Ana, CA, USA) in drinking water for 7 days, while mice in the control groups were given standard laboratory drinking water.

To confirm the association of gut microbiota and LPC, fecal microbiota transplantation (FMT) experiment was performed. Donors: WT mice and *Fut2*^△IEC^ mice were treated with 3% DSS for 7 days, and their feces were collected separately. Recipients: WT mice were randomly divided into two groups (FMT-WT, FMT-F) and were given a cocktail of antibiotics (100 mg/kg vancomycin, 200 mg/kg neomycin, 200 mg/kg metronidazole and 200 mg/kg ampicillin) every 12 h for 7 days by oral gavage (200ul per mouse) to prepare germ-free (GF) mice as previously described [21]. The feces from the donors were dissolved in sterile phosphate buffer saline (PBS) and centrifuged (500 g for 30 s) to prepare fecal bacteria suspension. The fecal suspensions were transplanted to GF mice (FMT-WT mice received the fecal suspensions from WT mice, and FMT-F mice received the fecal suspensions from *Fut2*^△IEC^ mice) by gavage once a day for 7 days. 1% DSS was given in drinking water during FMT for 7 days.

To investigate the role of Lysophosphatidylcholine (LPC) in colitis, WT mice were randomly divided into two groups (DSS + LPC group and DSS + PBS group). 1% DSS was given for 7 days. During the 7 days, mice in the two groups were respectively treated with LPC (40 mM, dissolved in PBS) (DSS + LPC group) and PBS (DSS + PBS group) everyday by enema.

### Histological examination

For histological examination, distal colon specimens were fixed in 4% formalin for 24 h and embedded in paraffin, stained with hematoxylin and eosin (H&E), and then analyzed by a pathologist without prior knowledge of experimental procedures. The histological analysis was calculated based on inflammation severity, inflammation extent and crypt damage as previously reported [22]. Disease activity index (DAI) scores were monitored every day to evaluate the severity of colitis, including weight loss, consistency of defecation and presence of bloody stools as described previously [23].

### Cell culture and treatment

Human epithelial colorectal adenocarcinoma (Caco-2) cells were cultured in Roswell Park Memorial Institute (RPMI) 1640 medium supplemented with 10% fetal bovine serum (FBS) and 1% penicillin–streptomycin. RAW264.7 cells were grown in Dulbecco's Modified Eagle Medium (DMEM) supplemented with 10% FBS and 1% penicillin–streptomycin. Cells were maintained in a 5% CO_2_ incubator at 37 °C. Cells were routinely tested to exclude mycoplasma contamination. During the experiment, Caco-2 cells were cultured for 2 weeks for further experiment. Drugs and concentrations included: Dimethylsulfoxide (DMSO) (0.5% vol/vol), LPC (50uM, Sigma, USA). Transepithelial/transendothelial electrical resistance (TEER) of Caco-2 cells was detected by Millicell ERS-2 Epithelial Volt-Ohm Meter (Millipore, MA, USA) following the manufacturer’s instructions and as previously described [24].

### ELISA analysis

Cell-free supernatants concentrations of TNF-α, IL-1β, serum concentration of lipopolysaccharide (LPS), and fecal concentration of LPC were detected by enzyme-linked immune sorbent assay (ELISA) (Neobioscience, Shenzhen, China; ELK Biotechnology CO.,LTD) according to the manufacturer's manuals. The absorbance was obtained by a microplate reader (Biotek Instruments, Inc., Winooski, VT, USA).

### Immunofluorescence and immunohistochemistry

For immunofluorescence staining, paraffin embedded sections (5 μm) with colon tissues were hydrated, treated for antigen retrieval with citrate buffer (pH 6), and the slides of cells were fixed in 4% formalin for 30 min and then washed with PBS for 3 times. Then they were blocked with 10% donkey serum for 30 min at 37 °C and incubated with the corresponding primary antibodies overnight at 4 °C. Antibodies used were: goat anti-ZO-1 (1:200, arigo) and rabbit anti-occludin (1:200, abcam). After washing with PBS for 3 times, slides were stained with secondary antibodies for 1 h at room temperature. Secondary antibodies used were Alexa Fluor 488 conjugated donkey anti-goat IgG and Alexa Fluor 594 conjugated donkey anti-rabbit IgG (1:200, Antgene Biotech Co., Ltd. Wuhan, China). For Ulex europaeus agglutinin-I (UEA-I) staining, sections were incubated with rhodamine UEA-I for 1 h at 37 °C. The nuclei were stained with DAPI (Beyotime Biotech, China) for 8 min at room temperature. Images were acquired on confocal microscope (Nikon, Japan).

Immunohistochemistry (IHC) of colon tissues was performed using a VECTASTAIN Elite ABC kit and a DAB Detection kit (Boster Biological Technology Co., Ltd) following the manufacturer’s instructions with an anti-F4/80 antibody (ARG55738, arigo).

### Mucous thickness and goblet cell number count

The colonic segments were fixed in Methanol-Carnoy solution, paraffin embedded and cut into serial 5 μm sections. Then, goblet cells and mucus were stained using Alcian blue/Periodic acid-Schiff (AB-PAS) staining methods as previously described [25].

### 16S rRNA sequencing and metabolomics analysis

Fecal microbial DNA was extracted from mice feces samples and subjected to 16S rRNA sequencing. The microbial composition and biodiversity were analyzed, in which alpha diversity was determined by chao1 index, and beta diversity was visualized by principal coordinate analysis (PCoA) based on Unweighted Unifrac. Besides, the relative abundance of specific family and genera were further identified. To obtain functions of fecal microbiota, functional microbial profiles were predicted using PICRUSt [26], a software for predicting functional abundance based on 16S rRNA sequence.

Untargeted metabolic profiling was performed by liquid chromatography-mass spectrometry (LC–MS) at Wuhan Metware Biotechnology Co, Ltd (Wuhan, China) as described previously [27, 28]. Metabolite data were log2-transformed for statistical analysis. Different metabolites were screened combining through fold change and VIP value. KEGG database was used for annotation of the different metabolites as described previously [29].

### Western blot analysis

Proteins were harvested from cells and colon tissues with RIPA Lysis Buffer (Beyotime, Hainan, Jiangsu, China) supplemented with phenylmethyl sulfonyl fluoride (PMSF) protease inhibitor and phosphatase inhibitor. Total protein concentration were determined by Pierce™ BCA Protein Assay Kit (Thermo Fisher, Waltham, Massachusetts, USA), denatured protein samples of appropriate quality of proteins were subjected to sodium dodecyl sulfate polyacrylamide gel electrophoresis (SDS-PAGE) and then transferred to PVDF membranes. Then membranes were later blocked with 5% skimmed milk, and incubated were immunodetected with specific antibodies against occludin ( 71–1500, Thermofisher, CA, USA), ZO-1 (ARG55738, Arigo, Taiwan, China), IL-1β (12242; Cell signaling Technology, Massachusetts, USA), TNF-α (a11534, Abclonal, Wuhan, China), and GAPDH (Antgene, Wuhan, China) overnight at 4 °C. The secondary antibody was purchased from GeneTex (Irvine, California, USA). Protein bands were visualized by the FluorChem Imaging System (ProteinSimple, San Jose, California, USA) using the commercial Pierce™ Fast Western Blot Kit and the ECL Substrate (Thermo Fisher, Waltham, Massachusetts, USA).

### RNA extraction and qPCR

RNA was extracted from colon tissue or cells using TRIzol reagent (Invitrogen) according to the manufacturer’s protocol. The reverse transcription (cDNA) was synthesized from 1 μg of total RNA with Prime Script RT Master Mix (Takara Biotechnology, Dalian, China). Quantitative Real-time PCR was performed using 1 μl first-strand cDNA with the LightCycler® 480 SYBR I Master Mix (Roche, Switzerland), in a final volume of 10 μl. All samples were run in triplicate and underwent 45 amplification cycles in a Roche LightCycler R480 (Roche, Switzerland). The relative fold change of mRNA expression was measured by using 2^−ΔCT^ method and normalized. Primers used can be seen on Additional file 1: Table S1.

### Statistical analysis

The SPSS 20.0, Graphpad prism software and Image J software were used for statistical analysis. Data were presented as mean values ± SEM for independent experiments. For comparison between two groups, a paired t-test was performed. Multiple group comparisons were calculated by one-way analysis of variance (ANOVA). The correlation between LPC and other indicators was assessed by Pearson correlation analysis. *P* < 0.05 was considered statistically significant.

## Results

### Fut2 and α-1,2-fucosylation are down-regulated in IBD patients and DSS-induced colitis mice

**Fig. 1:**
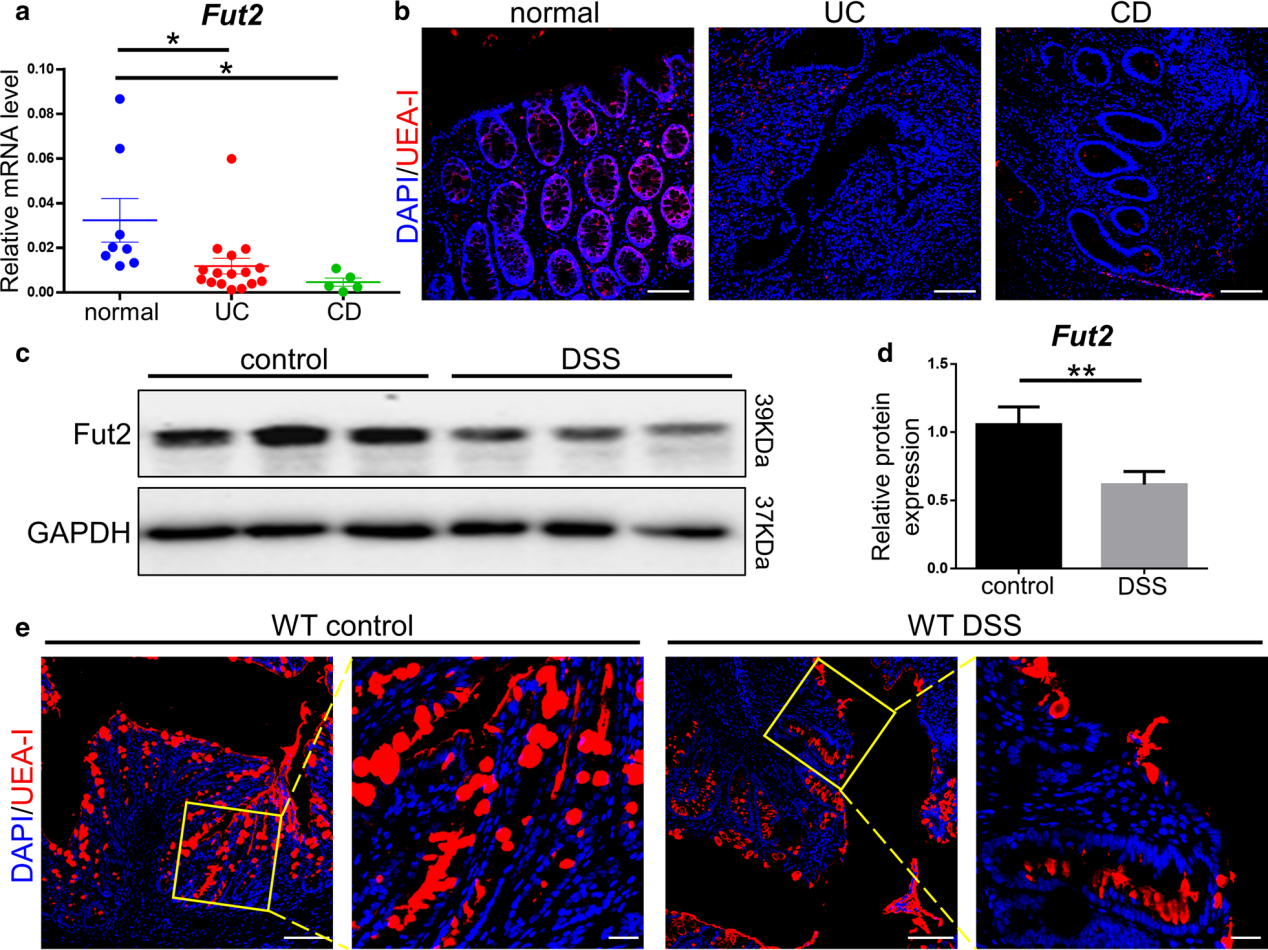
Down regulation of *Fut2* and α-1,2-fucosylation in IBD patients and DSS-induced mice. **a** The relative mRNA expression of *Fut2* in normal colon tissues and in that of UC, and CD patients was detected by qPCR (n = 8, 16, 5 for each group). **b** Typical UEA-I (a specific lectin for α-1,2-fucosylation) staining (red) images of normal, UC, and CD colon tissue (Scale bar, 100 μm). **c**, **d** The proteins level of *Fut2* in the colonic epithelial cells extracted from WT mice exposed or not exposed to DSS (n = 3 per group). **e** The typical images of mice colon tissues stained with UEA-I (Scale bar, 100 μm, 50 μm). Data are expressed as mean ± SEM. The data come from three independent experiments. In all panels: *p < 0.05, **p < 0.01. (*UEA-I* Ulex Europaeus Agglutinin-I)

In comparison of heathy individuals, the expression of *Fut2* gene was remarkably down-regulated in colon tissues of UC (*P* = 0.036) and CD patients (*P* = 0.031); *Fut2* in all 5 CD patients and in 15 out of 16 UC patients was lower than one standard deviation of the controls (Fig. 1a). UEA-I staining revealed that α-1,2-fucosylation was decreased in colonic epithelial of UC, CD patients (Fig. 1b). Besides, in mice exposed to DSS, the protein level of *Fut2* in colon tissues was significantly lower than that of normal mice (P = 0.009) (Fig. 1c, d). Decreased α-1,2-fucosylation in colon of mice exposed to DSS detected by UEA-I staining (Fig. 1e). The data above demonstrated that the expression of *Fut2* and α-1,2-fucosylation decreased in IBD patients and experimental colitis.

### Intestinal epithelium-specific Fut2 deficiency exacerbates DSS-induced colitis

**Fig. 2:**
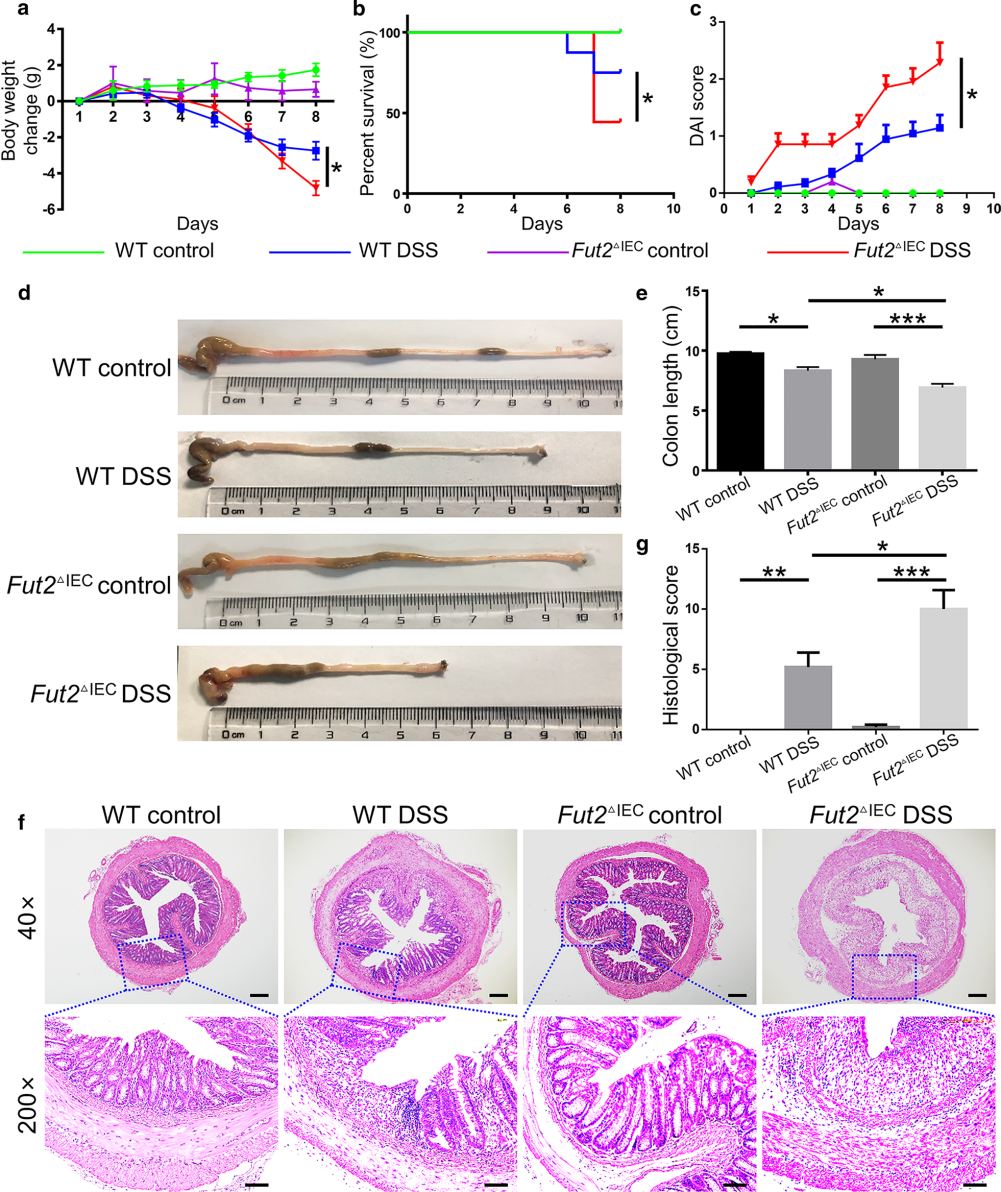
Intestinal epithelium-specific *Fut2* deficiency exacerbated DSS-induced colitis. **a** Body weight change during the disease process. **b** Survival rate of mice from each group. **c** Disease activity index evaluations of mice. **d**, **e** The length of colons from each group of mice. **f** Sections from colon tissue stained with H&E. **g** Histopathological scores of colons from each group of mice. Data are expressed as mean ± SEM (n = 5 per group).The data come from three independent experiments. In all panels: *p < 0.05, **p < 0.01, ***p < 0.001. (*DSS* dextran sulfate sodium, *H&E* hematoxylin and eosin)

Based on the decreased expression level of *Fut2* and α-1,2-fucosylation in IBD, we hypothesized a crucial functional relevance of *Fut2* in colitis. To evaluate the role of *Fut2* in colitis, we induced colitis in both WT and *Fut2*^△IEC^ mice by administering 3% DSS in drinking water for 7 days. *Fut2*^△IEC^ mice exhibited higher death rate (*P* = 0.036) than the WT mice when treated with DSS (Fig. 2b), accompanied by reduced colon length (*P* = 0.017) (Fig. 2a). And disease activity score of *Fut2*^△IEC^ mice was much higher compared to WT mice in DSS group (*P* = 0.024) (Fig. 2c). Besides, this was accompanied with shorter colon length (Fig. 2d, e). Consistent with the clinical signs, more severe glandular defects, mucosal ulceration and inflammatory cell infiltration in the colon sections (Fig. 2f) and higher histopathological scores (Fig. 2g) of *Fut2*^△IEC^ mice further confirmed the results. Collectively, intestinal epithelium-specific loss of *Fut2* exacerbates DSS-induced colitis.

### Fut2^△IEC^ mice exhibit increased inflammatory response

**Fig. 3:**
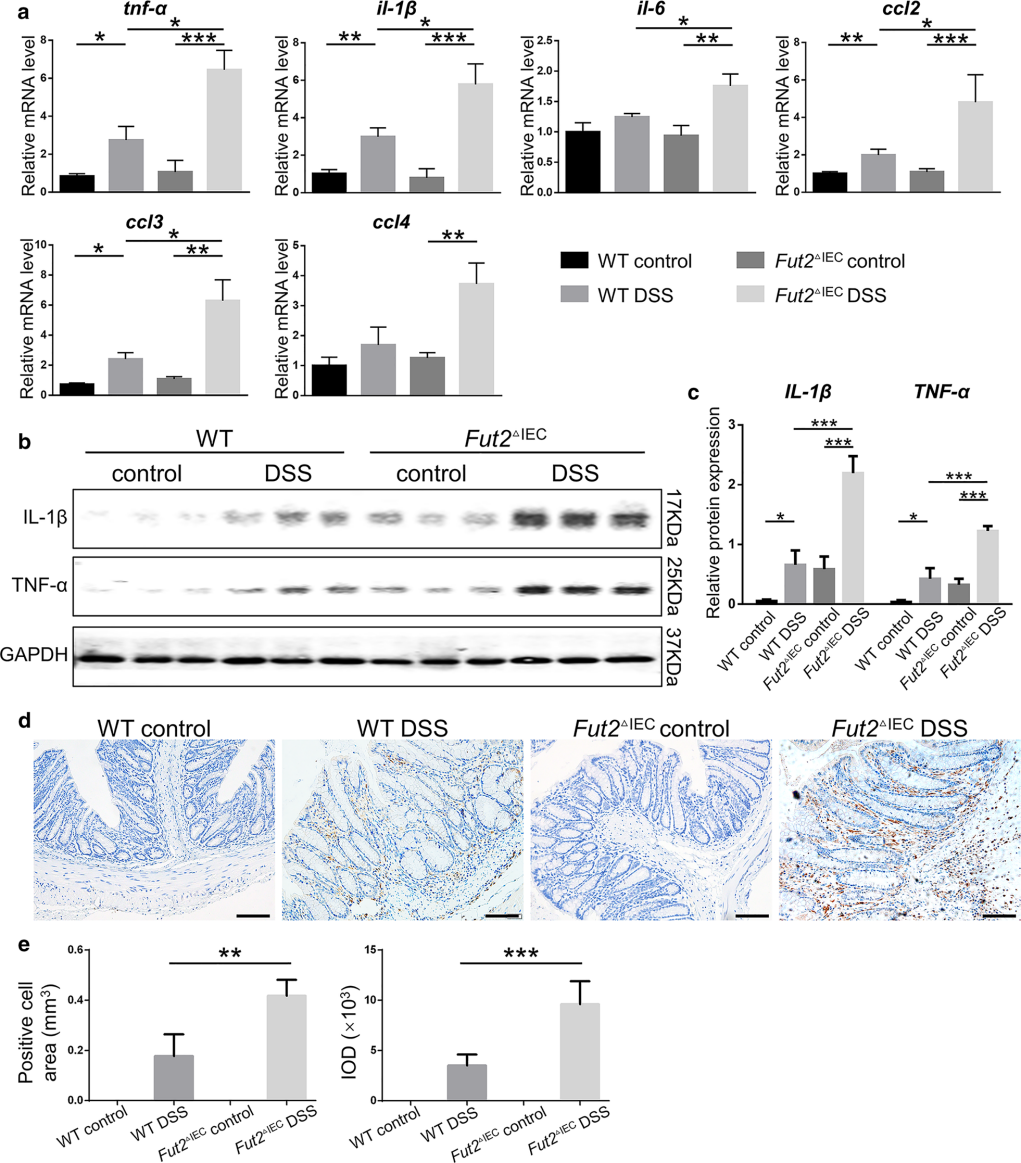
Loss of *Fut2* in colon epithelium increased the level of pro-inflammatory cytokines in colon following DSS exposure. **a** The relative mRNA expressions of *tnf-α*, *il-1β*, *il-6*, *ccl2*, *ccl3*, *ccl4*, in colon tissues were detected by qPCR. **b**, **c** The proteins level of TNF-α, IL-1β in colon tissues of mice in four groups. GAPDH was used as a loading control. **d**, **e** Immunohistochemistry of F4/80 + for macrophages in the colon tissues of mice in each group. (Scale bar, 100 μm) The results were analyzed by analysis of positive cells area and DOI. Data are expressed as mean ± SEM (n = 5 per group).The data come from three independent experiments. In all panels: *p < 0.05, **p < 0.01, ***p < 0.001

Inflammatory response is a crucial indicator for the evaluation of colitis, therefore intestinal pro-inflammatory cells and factors were detected. Mice in WT control group and *Fut2*^△IEC^ control group showed no difference in pro-inflammatory factors in colon tissues, however, the *Fut2*^△IEC^ mice exposed to DSS showed upregulated mRNA levels of pro-inflammatory factors associated with macrophages such as *tnf-α* (*P* = 0.028)*, il-1β* (*P* = 0.002)*, il-6* (*P* = 0.030) in colon tissues compared with WT mice. Besides, *ccl2* (*P* = 0.026)*, ccl3* (*P* = 0.031), *the* chemokines associated with monocytes/macrophages were increased as well (Fig. 3a). This result was further confirmed by western blot of IL-1β (*P* = 0.002) and TNF-α expression (*P* = 0.0021) (Fig. 3b, c). Immunohistochemistry staining of F4/80 was done to evaluate colonic macrophages infiltration (Fig. 3d, e). It showed that the macrophages (F4/80 +) were 3 times more in *Fut2*^△IEC^ DSS group compared with WT DSS group (*P* = 0.001). Collectively, these results demonstrated that *Fut2* deficiency increased inflammatory response in DSS induced acute colitis.

### Loss of intestinal epithelium-specific Fut2 aggravates the intestinal barrier damage in DSS-induced colitis

**Fig. 4:**
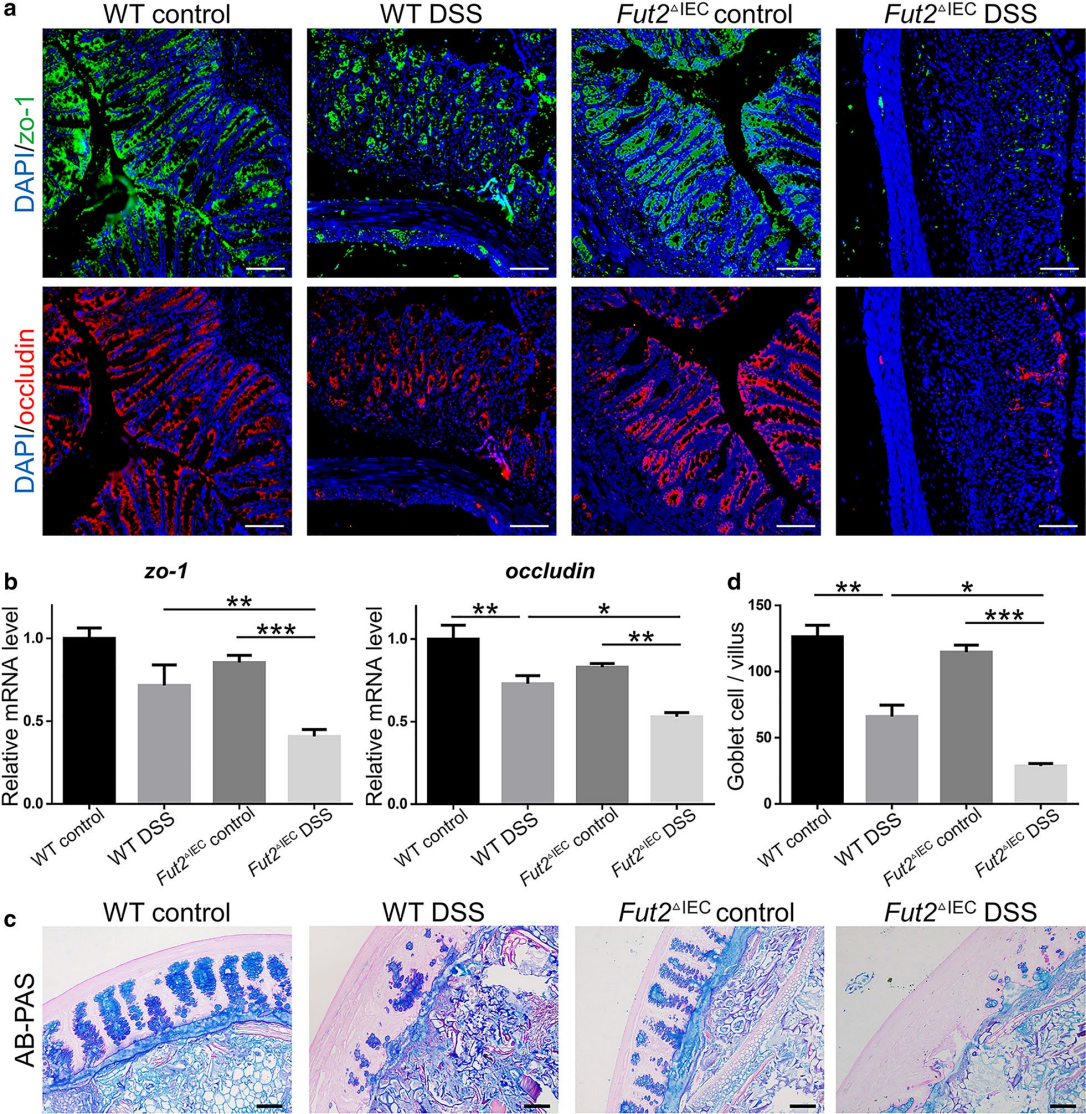
*Fut2* deficiency aggravated the epithelial tight junction and mucus barrier damage in DSS-induced colitis. **a** The typical immunofluorescent images of colon tissues stained with ZO-1 (green), occludin (red), the principal components of tight junction (Scale bar, 100 μm) **b** Relative mRNA expression of *occludin* and *ZO-1* in different groups. **c** The typical histological images of colon tissue stained with AB/PAS from different groups (Scale bar, 100 μm). **d** The number of goblet cells (Scale bar, 50 μm). Data were shown as mean ± SEM; (n = 5 per group). The data come from three independent experiments. In all panels: *p < 0.05, **p < 0.01, ***p < 0.001

The severity of colitis is also correlated with the severity of epithelial barrier injury. Tight junction is one of the most important elements of epithelial barrier, and secretion of goblet cells plays an vital part in epithelial barrier. There was significant destruction of epithelial tight junction in *Fut2*^△IEC^ DSS group than that in the other 3 groups. The mRNA levels of *ZO-1* and *occludin* (the important components of tight junction) were found to be remarkably (*P* = 0.021and *P* = 0.047 respectively) decreased in *Fut2*^△IEC^ mice than WT mice when exposed to DSS (Fig. 4b). Immunofluorescent staining of ZO-1 and occludin confirmed this result (Fig. 4a). Furthermore, AB-PAS staining (Fig. 4c) of the colon tissues showed significantly decrease of mucous thickness and numbers of goblet cells (*P* = 0.013) (Fig. 4d) in *Fut2*^△IEC^ mice compared with WT mice in DSS-induced colitis. Briefly, *Fut2* deficiency in intestinal epithelium aggravates the intestinal barrier injury in DSS-induced colitis.

### The gut microbiota is structurally and functionally altered and the level of LPC is elevated in Fut2^△IEC^ mice

**Fig. 5:**
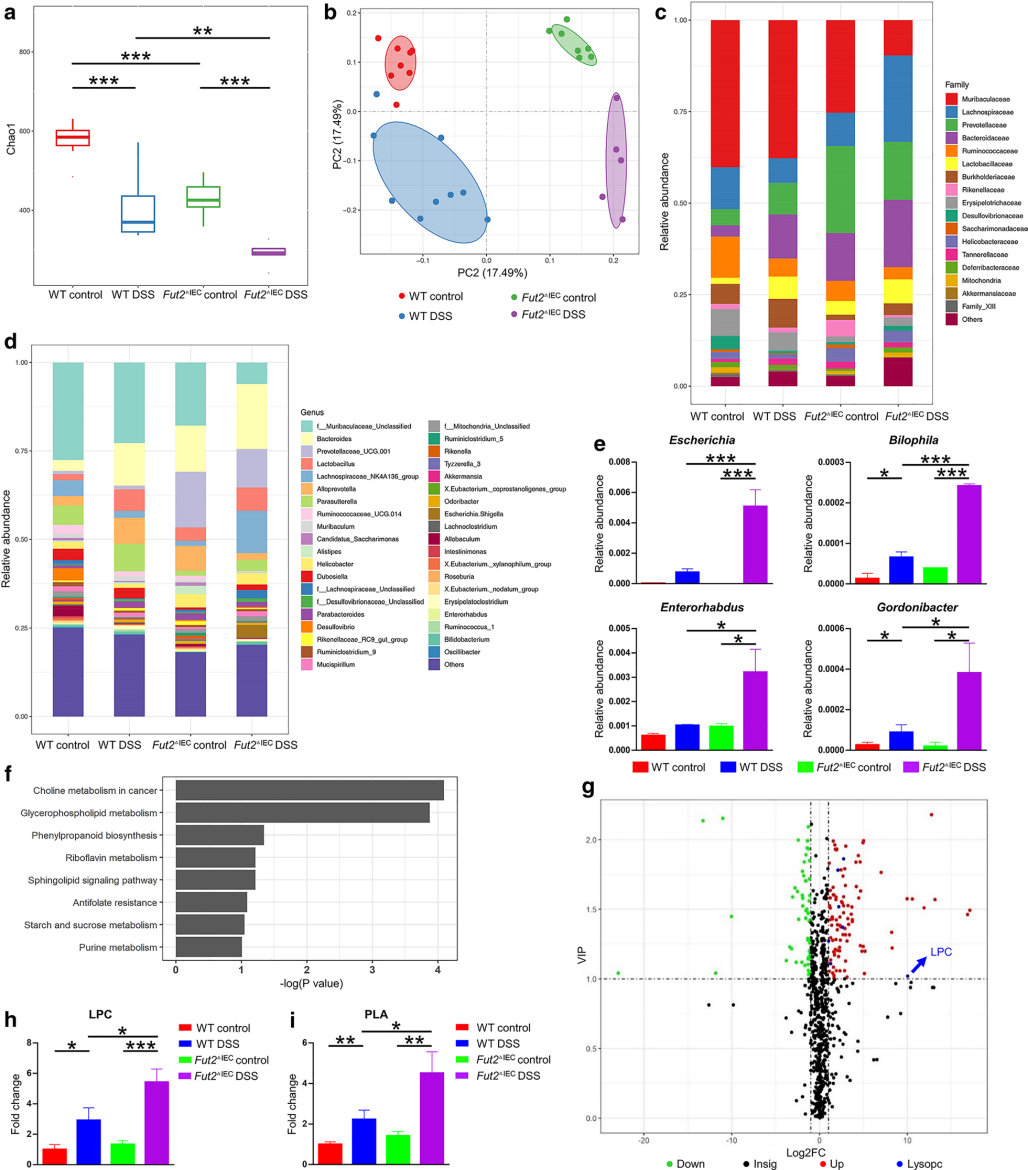
Altered gut microbiota and metabolism in Fut2^△IEC^ mice. **a** The alpha diversity (Chao1 index) in four groups. **b** The PCoA plots of unweighted UniFrac distances of beta diversity. **c**, **d** Relative abundance of microbial family and genus in different groups. **e** Relative abundance of microbiota at the genus level. **f** Increased metabolite of Fut2^△IEC^ versus WT mice when exposed to DSS. **g** Volcano Plot of differential metabolites in WT mice and Fut2^△IEC^ mice in DSS group (Fut2^△IEC^ mice versus WT mice). **h** The level of fecal LPC in four groups. **i** The level of PLA in four groups. Data were shown as mean ± SEM (n = 5 per group). The data come from three independent experiments. In all panels: *p < 0.05, **p < 0.01, ***p < 0.001. (*LPC* Lysophosphatidylcholine, *PLA* Phospholipase A)

Since difference between the WT mice and *Fut2*^△IEC^ mice was detected as depicted above, we explored the potential factors that mediated the difference in susceptibility to colitis. As mentioned before, *Fut2* has potential regulatory effect on gut microbiota, thus we speculated that gut microbiota may be one important factor affecting colonic injury in *Fut2*^△IEC^ mice. In the first step, we explored the composition of the microbiota at baseline and during colitis using 16S diversity analysis of the mice feces. *Fut2*^△IEC^ mice exhibited lower alpha-diversity (Chao1 index) than WT mice (p < 0.001). When exposed to DSS, the Chao1 index deceased more significantly in *Fut2*^△IEC^ mice than in WT mice (Fig. 5a). The PCoA plot based on Unweighted Unifrac (Fig. 5b) indicated a significant separation among the microbiota of the four groups. As it is shown in Fig. 5c, there were significant reduction in the relative abundance of *Muribaculaceae* family and *Ruminococcaceae* family in *Fut2*^△IEC^ mice treated with DSS*.* At the genus level, the relative abundance of *bacteroides* was increased in *Fut2*^△IEC^ mice treated with DSS compared with the other three groups (Fig. 5d).

Since composition of gut microbiota is closely related to its function, we next performed nontargeted metabolomics analysis of the feces to investigate function of gut microbiota. Notably, the metabolomics analysis revealed that the choline metabolism and glycerophospholipid metabolism pathways were significantly enhanced in *Fut*2^△IEC^ DSS feces compared to that in WT DSS feces (Fig. 5f). These data suggested that gut microbial metabolic function may differ between WT mice and *Fut*2^△IEC^ mice. We further analyzed the metabolites in the two enhanced pathways and found elevated level of LPC in *Fut*2^△IEC^ DSS mice compared with WT mice (Fig. 5g, h). Besides, PICRUST2 analysis indicated that Phospholipase A (PLA), the microbial enzyme responsible for the production of LPC, was markedly enriched in *Fut2*^△IEC^ mice subjected to DSS (Fig. 5i). Correlation analysis revealed that the concentration of LPC was positively correlated with four gram-negative bacteria—*Escherichia, Bilophila, Enterorhabdus* and *Gordonibacter* (Table 1 and Additional file 1: Figure S2c). The relative abundance of these bacteria were increased in *Fut2*^△IEC^ DSS mice (Fig. 5e).

**Table 1:** Correlation analysis

	R^2^	Pearson correlation coefficient (r)	*P* value
LPC & *tnf-α*	0.622	0.789	0.008
LPC & *il-1β*	0.2445	0.494	0.072
LPC & *il-6*	0.5442	0.738	0.002
LPC & *ccl2*	0.7737	0.880	< 0.001
LPC & *ccl3*	0.7592	0.871	< 0.001
LPC & *occludin*	0.3815	-0.593	0.025
LPC & *ZO-1*	0.1403	-0.375	0.187
LPC &Escherichia	0.8844	0.9404	< 0.001
LPC &Bilophila	0.8399	0.9164	< 0.001
LPC &Enterorhabdus	0.7547	0.8688	< 0.001
LPC &Gordonibacter	0.8309	0.9115	< 0.001

Correlation analysis showed that LPC concentration was positively correlated with pro-inflammatory cytokines and negatively correlated with the expression of tight junction proteins (see in Table 1 and Additional file 1: Figure S2a, b).

Collectively, these data demonstrated that structure and some metabolic pathways of gut microbiota differed between WT and *Fut2*^△IEC^ mice, and more LPC was generated in *Fut2*^△IEC^ mice than WT mice when exposed to DSS.

### Gut microbiota from *Fut2*^*△IEC*^* mice* promotes the generation of LPC, aggravating colitis

**Fig. 6:**
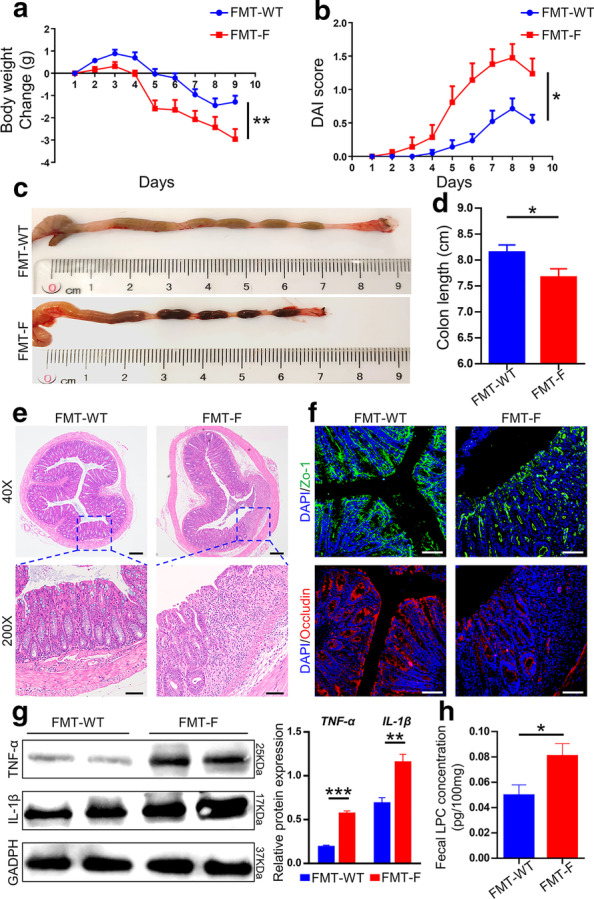
Gut microbiota from *Fut2*^*△IEC*^* mice* promotes the generation of LPC, aggravating colitis **a** Body weight change of mice in FMT-WT group and FMT-F group during the process. **b** DAI evaluations for the two groups. (C,D) The length of colons from each group of mice. **e** Representative H&E staining images of colonic sections. **f** Immunofluorescent images of colon tissues stained with ZO-1 (green), and occludin (red) in each group (Scale bar, 100 μm). **g** The proteins level of TNF-α, IL-1β in colon tissues. GAPDH was used as a loading control. (H) Fecal concentration of LPC of mice in each group. Data are expressed as mean ± SEM (n = 5 per group). The data come from three independent experiments. In all panels: *p < 0.05, **p < 0.01, ***p < 0.001. (*LPC* lysophosphatidylcholine, *H&E* hematoxylin and eosin)

To confirm that generation of LPC was associated with gut microbiota, FMT was performed. As shown in Fig. 6a, mice (FMT-F group) received the fecal microbiota from *Fut2*^*△IEC*^ mice exhibited more weight loss than the mice (FMT-WT group) received the fecal microbiota from WT mice (*P* = 0.009). Higher DAI scores (Fig. 6b) (*P* = 0.014) and reduced colon length (*P* = 0.049) (Fig. 6c, d) were observed in FMT-F group. H&E staining of the colon sections revealed more severe colitis in FMT-F mice than FMT-WT mice pathologically. Besides, immunofluorescence staining indicated that fecal microbiota from *Fut2*^*△IEC*^ mice reduced the expression of ZO-1 and occludin in the colon compared with the other group (Fig. 6f). The protein levels of the proinflammatory factors TNF-α (*P* = 0.0005) and IL-1β (*P = *0.0012) were detected to be higher in the FMT-F group by Western blotting (Fig. 6g). Most importantly, the fecal LPC concentration of FMT-F mice was higher than that of FMT-WT mice (*P* = 0.042, Fig. 6h), which confirmed the association of LPC and gut microbiota.

### LPC exacerbates DSS-induced colitis

**Fig. 7:**
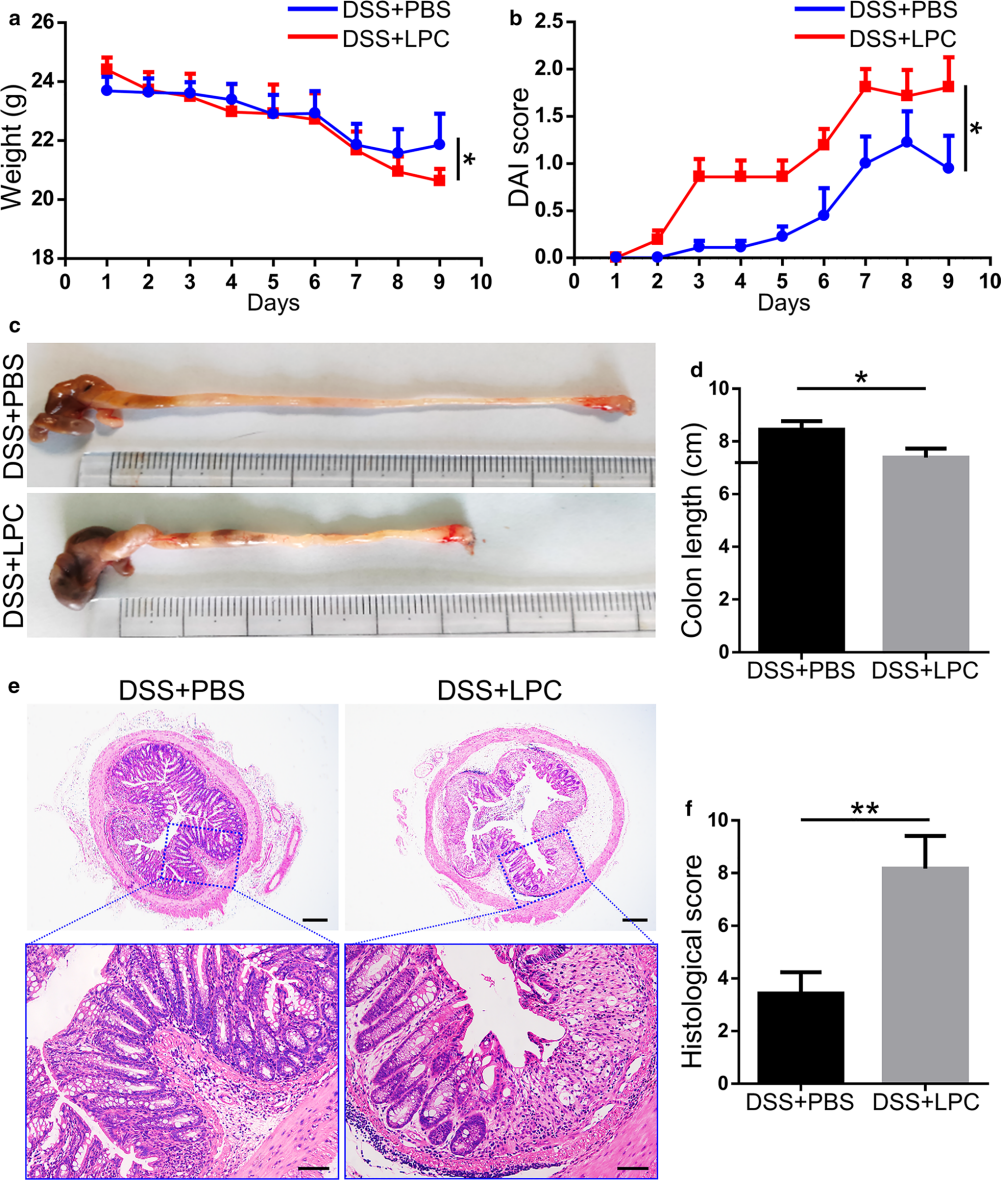
LPC exacerbated DSS-induced colitis. **a **Body weight change of mice during the disease process. (B) Disease activity index (DAI) evaluations of mice in two groups. **c**, **d** The length of colons from each group of mice. (E) Serial sections from colon tissues stained with H&E. **f** Histopathological scores of colons in mice of each group. Data are expressed as mean ± SEM (n = 6 per group). The data come from three independent experiments. In all panels: *p < 0.05, **p < 0.01, ***p < 0.001. (*LPC* lysophosphatidylcholine, *DSS* dextran sulfate sodium, *H&E* hematoxylin and eosin)

To determine whether LPC could exacerbated DSS-induced colitis, WT mice were randomly divided into two groups. Both groups were exposed to 1% DSS for 7 days in drinking water, and mice in the DSS + LPC group were also given LPC (40 mM, dissolved in PBS) by enema once a day for 7 days and the DSS + PBS group were given PBS as control. As shown in Fig. 7a, DSS + LPC mice exhibited significantly more weight loss at the end of the experiment (P = 0.047) compared with those of mice subjected to DSS only. Higher DAI scores (Fig. 7b) (*P* = 0.025) and shorter colon length (*P* = 0.047) (Fig. 7c, d) were observed in DSS + LPC group. Histopathologic analysis (Fig. 7e, f) (*P* = 0.007) of colon tissues confirmed the results that LPC exacerbated DSS-induced colitis. Collectively, LPC exacerbated DSS-induced colitis.

### LPC increases inflammatory response in vitro and in vivo

**Fig. 8:**
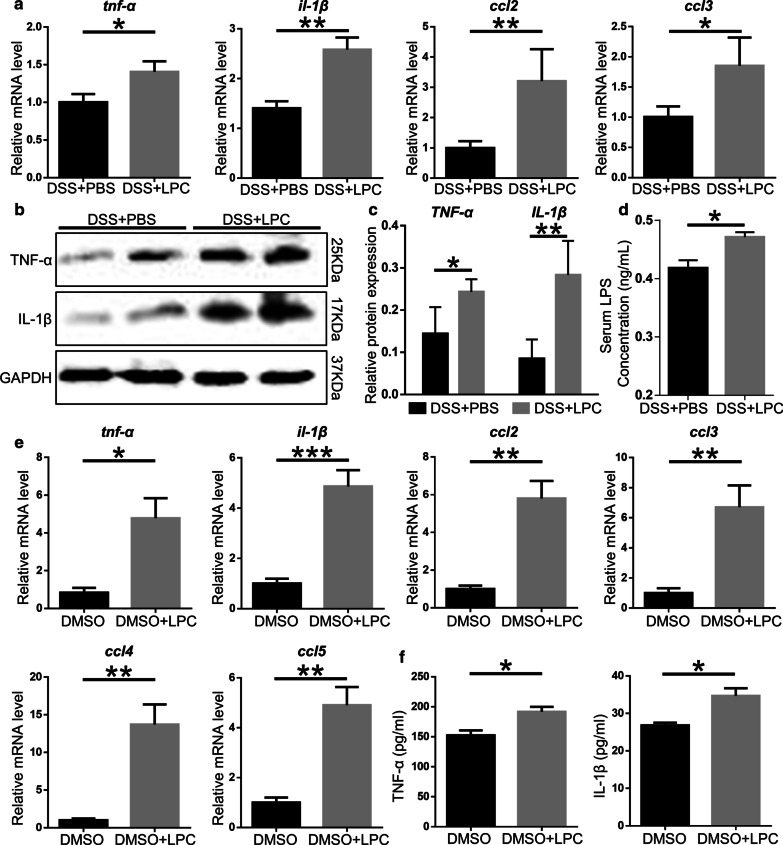
LPC increase pro-inflammatory cytokines in vitro and in vivo*.* **a** The relative mRNA expression of *tnf-α, il-1β, ccl2, ccl3* in colon tissues was detected by qPCR (n = 6 per group). **b**, **c** The proteins level of TNF-α, IL-1β in colon tissues. GAPDH was used as a loading control. **d** Serum LPS levels. **e** The relative mRNA expression of *tnf-α*, *il-1β*, *ccl2*, *ccl3*, *ccl4* and *ccl5* in DMSO and DMSO + LPC group (n = 5 per group). **f** The level of TNF-α and IL-1β in culture supernatant of Raw 264.7 cells in the presence or absence of LPC was detected by ELISA (n = 5 per group). Data are expressed as mean ± SEM. The data come from three independent experiments. In all panels: *p < 0.05, **p < 0.01, ***p < 0.001

Inflammatory response is an important index to evaluate the severity of colitis, so we tested the mRNA and protein level of pro-inflammatory cytokines in colon tissues of mice subjected to DSS + PBS and DSS + LPC. The mRNA expression of *tnf-α* (*P* = 0.046)*, il-1β* (P = 0.002)*, ccl2* (*P* = 0.006)*,* and *ccl3* (*P* = 0.019) increased in mice subjected to LPC (Fig. 8a). The protein expression of TNF-α (*P* = 0.021), IL-1β (*P* = 0.006) in DSS + LPC mice was 2–3 times higher than that in DSS + PBS mice (Fig. 8b, c). Besides, the serum level of LPS was increased in DSS + LPC mice compared to DSS + PBS mice (P = 0.011, Fig. 8c). In vitro, Raw 264.7 cells treated with LPC (50uM) for 12 h exhibited significant increase of pro-inflammatory cytokines including *tnf-α, il-1β, ccl2, and ccl3* compared to cells treated without DMSO. The mRNA level of *ccl4* (*P* = 0.001) *and ccl5* (P = 0.002) was also detected to be much higher in the DMSO + LPC group (Fig. 8e). Meanwhile, Raw 264.7 cells treated with LPC secreted more pro-inflammatory cytokines TNF-α (*P* = 0.015) and IL-1β (*P* = 0.004) in supernatant which was tested by ELISA (Fig. 8f). In short, LPC promoted colon inflammation response*.*

### LPC impairs tight junctions in vitro and in vivo

**Fig. 9:**
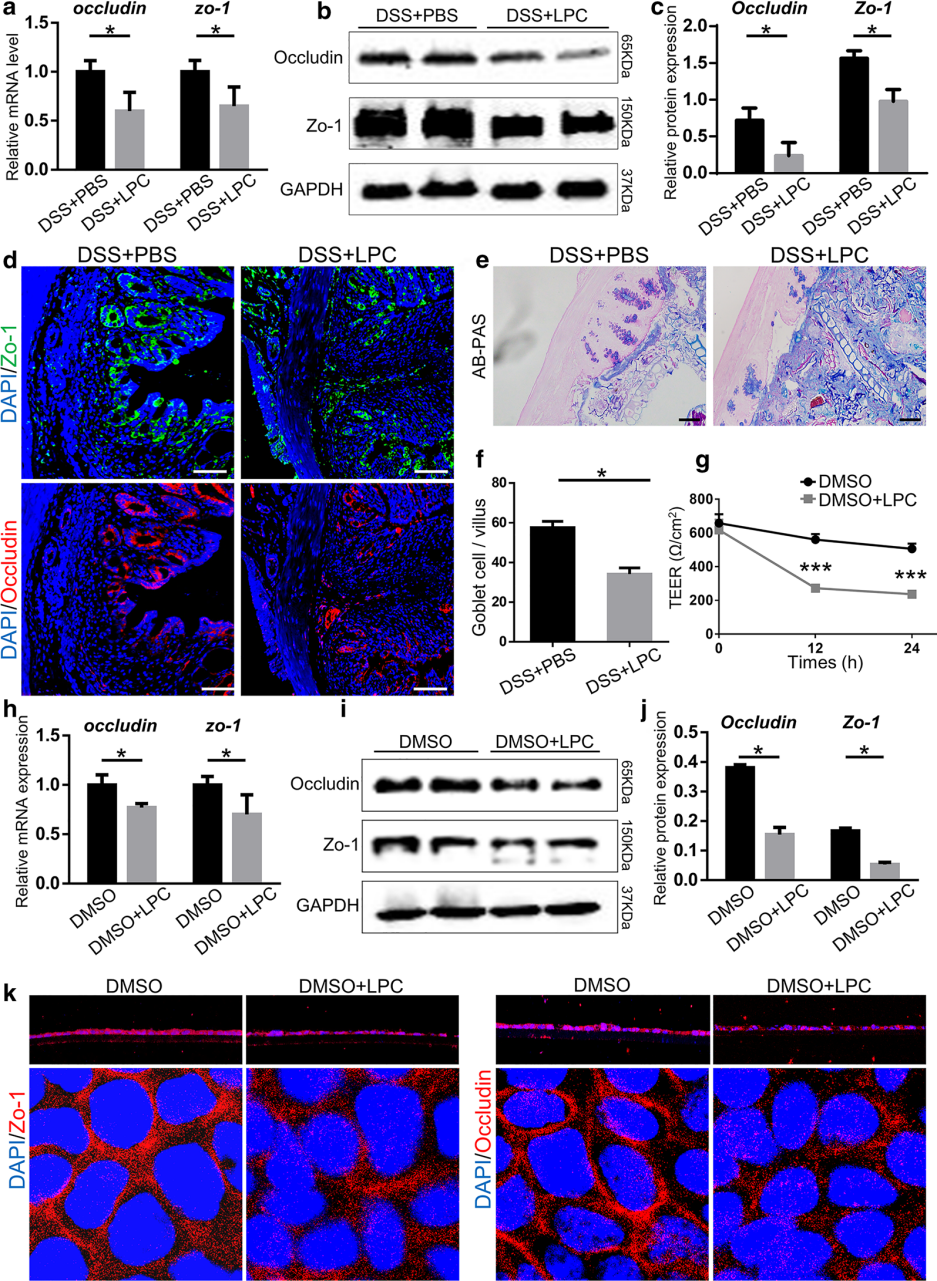
LPC impaired intestinal barrier function in vitro and in vivo*.* **a** Relative mRNA and **b**, **c** Protein expression of occludin and ZO-1 in colon tissues. GAPDH was used as a loading control. **d** The typical immunofluorescent images of colon tissues stained with ZO-1 (green), and occludin (red) in each group (Scale bar, 100 μm). **e**, **f** The typical images of colon tissue stained with AB/PAS and the number of goblet cells from two groups (Scale bar, 100 μm). **g** TEER of Caco-2 cells treated or not treated with LPC. (H) The mRNA level of *ZO-1* and *occludin* in caco-2 cells treated with LPC of two groups. **i**, **j** The protein levels of ZO-1 and occludin in caco-2 cells treated with LPC of two groups. **k** The immunofluorescent images of Caco-2 cells with ZO-1 and occludin in each group (Z-axis and XY-axis). Data were shown as mean ± SEM (n = 6 per group). The data come from three independent experiments. In all panels: *p < 0.05. (*AB/PAS* alcian blue/periodic acid Schiff, *TEER* transepithelial/transendothelial electrical resistance)

To further determine the effect of LPC on the integrity of epithelial barriers, the mRNA and protein levels of ZO-1 and occludin were detected to be significantly lower in DSS + LPC mice compared with those in DSS + PBS mice (Fig. 9a–c). Immunofluorescent staining of colon tissues demonstrated the expression of ZO-1 and occludin were impaired by LPC (Fig. 9d). The number of goblet cells was significantly decreased in DSS + LPC mice compared to DSS + PBS mice, so was the mucus thickness (Fig. 9e, f). Correspondingly, in vitro, the TEER of two groups –the DMSO group and the DMSO + LPC group – were measured in Caco-2 cells at different time points (0, 12, 24 h). At the beginning of the experiment, no differences were detected between the two groups. However, after 12 and 24 h treatments, the TEER of the DMSO + LPC group was significantly decreased compared with that of the DMSO group (*P* = 0.0003 and 0.0006, respectively) (Fig. 9g). Moreover, the mRNA and protein levels of *ZO-1* and *occludin* in caco-2 cells treated with LPC were some half of the other group (Fig. 9h, i, j). Immunofluorescence of ZO-1 and occludin exhibited that tight junction of caco-2 cells was damaged when treated with LPC (Fig. 9k). All the data above demonstrated that LPC could impaired intestinal barrier functions.

## Discussion

In the present study, decreased *Fut*2 expression and α-1,2-fucosylation in colon were observed in patients with UC and CD. Moreover, we demonstrated that *Fut*2 deficiency in intestinal epithelium exacerbated colitis, including promoting release of pro-inflammatory cytokines and aggravating epithelial barrier damage. Gut bacterial diversity and composition altered in *Fut2*^△IEC^ mice and the generation of LPC was increased when exposed to DSS. FMT experiment confirmed that gut microbiota contributes to the generation of LPC, promoting colitis. Correlation analysis showed that LPC was positively correlated with pro-inflammatory cytokines and negative with expression of tight junction proteins. Finally, LPC was proved to increase pro-inflammatory cytokines and damage epithelial barrier in *vivo* and in *vitro* in our research. The role of *Fut2* in IBD susceptibility was to modulate gut microbiota structurally and functionally, thus altering generation of LPC which was proved to exacerbate colitis.

In our study, we revealed down regulation of  α-1,2-fucosylation in colonic epithelium in both of patients with CD and UC. Existing studies have reported the possible correlation between *Fut2* and CD susceptibility but no reports in the cases of UC. According to our findings, *Fut2* might be a risk genetic locus not only in CD but also in UC. The changes of gene in intestinal epithelial cells may alter the function of the cells themselves, influence the crosstalk between gut and other organs, or affect gut microbiota. Since one important function of *Fut2* is to promote the synthesis of fucosylated oligosaccharide chain on the intestinal mucosa which can act as both attachment sites and carbon source for intestinal bacteria [30–34], gut microbiota was studied in our research.

In comparison of WT control mice, gut microbiota diversity was declined in *Fut2*^△IEC^ mice without DSS. Tong M et al. [12] found that *Fut2* was associated with the composition of gut microbiota. They analyzed lavage samples collected from the cecum and sigmoid colons of 39 healthy subjects (12 SeSe, 18 Sese and 9 sese) and they found that the colonic microbiota of non-secretors (sese) was altered. Thought the gut microbiota was changed, in our study, no changes of colonic inflammation and barrier function in WT and *Fut2*^△IEC^ mice without DSS was showed. Therefore, we want to know whether *Fut2*^△IEC^ mice were susceptible to experimental DSS-induced colitis.

When treated with DSS, gut microbiota diversity of *Fut2*^△IEC^ mice was significantly lower than that of WT mice. And the composition was also altered, especially the decrease in the beneficial members of the gut community, like the Ruminococcaceae and Muribaculaceae family in *Fut2*^△IEC^ mice than WT mice. Moreover, FMT experiment confirmed that gut microbiota from *Fut*2^△IEC^ mice increase the susceptibility to colitis. This was the first time to study the changes of *Fut2*^△IEC^ mice in a DSS model. The perturbation of intestinal microbiota played an important role in the aggravation of intestinal damage in *Fut2*^△IEC^ mice.

It is accepted that composition of intestinal microbiota is closely associated with its function, and thereby may exert effects on host metabolism [18, 20]. In our study, differential metabolite analysis revealed that a metabolite, LPC, was increased obviously in *Fut*2^△IEC^ DSS mice compared with the other three groups. Besides, our FMT experiment disclosed that gut microbiota was associated with the LPC. The role of LPC on macrophages in atherosclerosis has been extensively studied [35–38], but few has been reported about its effects on colon. In our study, we detected macrophage-associated pro-inflammatory cytokines in mice treated with LPC, and we revealed LPC could promote the release of pro-inflammatory cytokines in colon tissue. In *vitro*, Raw 264.7 cells treated with LPC were detected to release more pro-inflammatory cytokines as well. Besides, C Tagesson et al. used a rat experimental model to evaluate the effect of LPC on intestinal permeability, and they found that LPC may impair the ileum mucosal cells [39]. In our study, tight junction of colonic epithelial cells treated with LPC was found to be damaged in *vivo* and in *vitro*. LPC exacerbated colonic inflammation and epithelial barrier damage in *Fut*2^△IEC^ mice. If the production of LPC could reduced, the inflammation and epithelial barrier damage in colon will be alleviated.

Certainly, there are inherent limitations of the present study that should be considered. Thought we found that *Fut2* was decreased in CD and UC, it will be more convinced if more data about human were included. So next step human experiments will be carried out to validate our results.

## Conclusion

In conclusion, our present study, for the first time, constructed *Fut2*^△IEC^ mice for the research on the role of *Fut2* in IBD. We demonstrated that intestinal epithelium-specific *Fut2* deficiency increase susceptibility to IBD through modulation of gut microbiota and generation of LPC. These data provide direction for future studies designed to increase intestinal fucosylation or reduce LPC production in IBD, especially in those with *Fut2* gene defect.

## Supplementary Information

**Additional file 1. **Additional tables and figures.

## Data Availability

The datasets during and/or analyzed during the current study available from the corresponding author on reasonable request.

## Abbreviations

CD: Crohn’s disease
DSS: Dextran sulfate sodium
DAI: Disease activity index
DMEM: Dulbecco's Modified Eagle Medium
fut: Fucosyltransferase
FBS: Fetal bovine serum
FMT: Fecal microbiota transplantation
GWAS: Genome wide association study
H&E: Hematoxylin and eosin
IBD: Inflammatory bowel disease
LC–MS: Liquid chromatography-mass spectrometry
LPC: Lysophosphatidylcholine
LPS: Lipopolysaccharide
PBS: Phosphate buffer saline
PSC: Primary sclerosing cholangitis
PLA1: Phospholipase A1
PCoA: Principal coordinate analysis
TEER: Transepithelial/transendothelial electrical resistance
UEA-I: Ulex europaeus agglutinin-I
UC: Ulcerative colitis
