# Supplementary material for: Gut microbiota-mediated lysophosphatidylcholine generation promotes colitis in intestinal epithelium-specific Fut2 deficiency
Source: J Biomed Sci. 2021 Mar 15;28:20. doi: 10.1186/s12929-021-00711-z (PMC7958775; doi:10.1186/s12929-021-00711-z)
Supplement: Supplementary file 1 — Additional file 1. Additional tables and figures. [file 12929_2021_711_MOESM1_ESM.pdf]

Supplementary Table.1

Gene	Forward primer (5′- 3′)	Reverse primer(5′- 3′)
human		
Gapdh	CCCCACTTGATTTTGGAGGGA	AGGGCTGCTTTTAACTCTGGT
Fut2	CTACCACCTGAACGACTGGATG	AGGGTGAACTCCTGGAGGATCT
ZO-1	CCATTCCCGAAGGAGTTGA	CGACGAGGAGTCGGATGATTT
occludin	CTCTCAGCCAGCCTACTCTTT	CATAGCCATAGCCACTTCCGT
mice		
gapdh	CATGGCCTTCCGTGTTCCTA	TACTTGGCAGGTTTCTCCAGG
fut2	ACCACAGCCAGAAGAGGATTG	GAAAGGTACCTGGGCACTCG
tnf-α	CATCTTCTCAAAATTCGAGTGACAA	TGGGAGTAGACAAGGTACAACCC
ccl2	TTGGCTCAGCCAGATGCA	CCTACTCATTGGGATCATCTTGC
il-1β	CCGTGGACCTTCCAGGATGA	GGGAACGTCACACACCAGCA
il-6	AGGATACCACTCCCAACAGACCT	CAAGTGCATCATCGTTGTTCATAC
cxcl-1	TGCACCCAAACCGAAGTCATAGCC	AGCCAGCGTTCACCAGACAGGT
ccl3	CCATGACACTCTGCAACCAAG	ACGATGAATTGGCGTGGAAT
ccl4	CCTCCCACTTCCTGCTGTTT	GCTTGGAGCAAAGACTGCTG
ccl5	GCCCACGTCAAGGAGTATTTC	CACACACTTGGCGGTTCCTT
ZO-1	GCTTTAGCGAACAGAAGGAGC	TTCATTTTTCCGAGACTTCACCA
occludin	TGAAAGTCCACCTCCTTACAGA	CCGGATAAAAAGAGTACGCTGG

Supplementary Figure 1

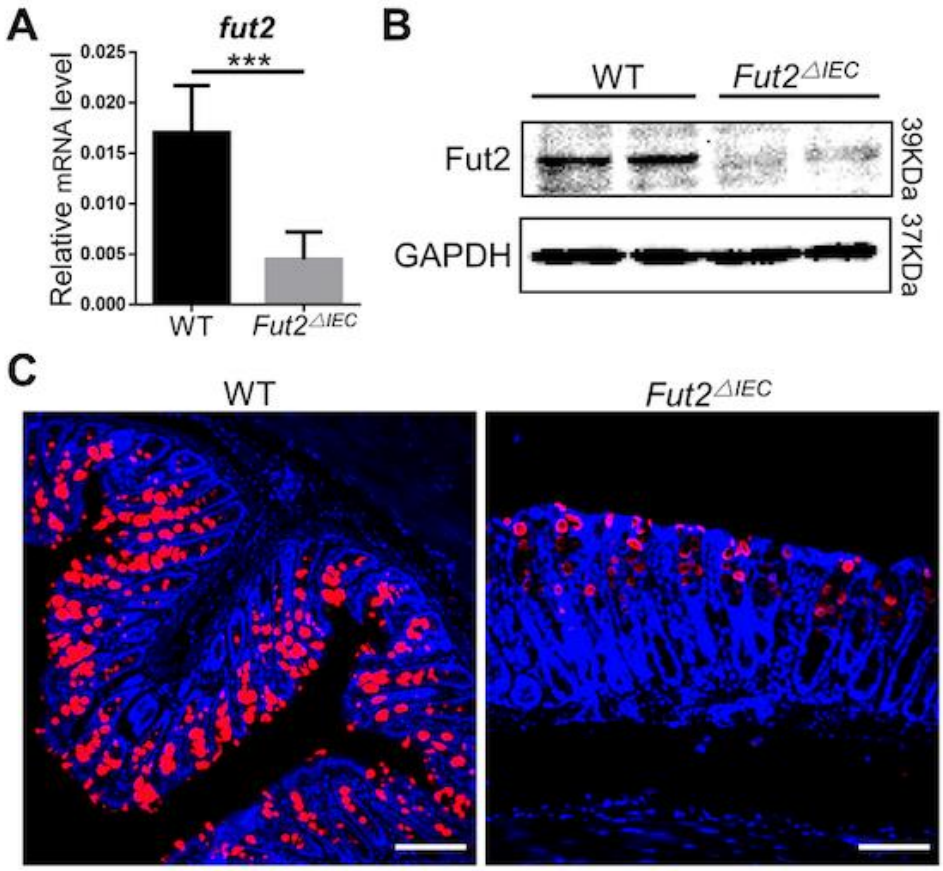

Supplementary Figure 1. (A) The mRNA level of *Fut2* in colonic epithelial cells of WT and *Fut2*$^{\triangle IEC}$ mice. (B) The proteins level of *Fut2* in colonic epithelial cells of WT and *Fut2*$^{\triangle IEC}$ mice. GAPDH was used as a loading control. (C) UEA-I staining (red) images of colon tissue in WT and *Fut2*$^{\triangle IEC}$ mice (Scale bar, 100μm).

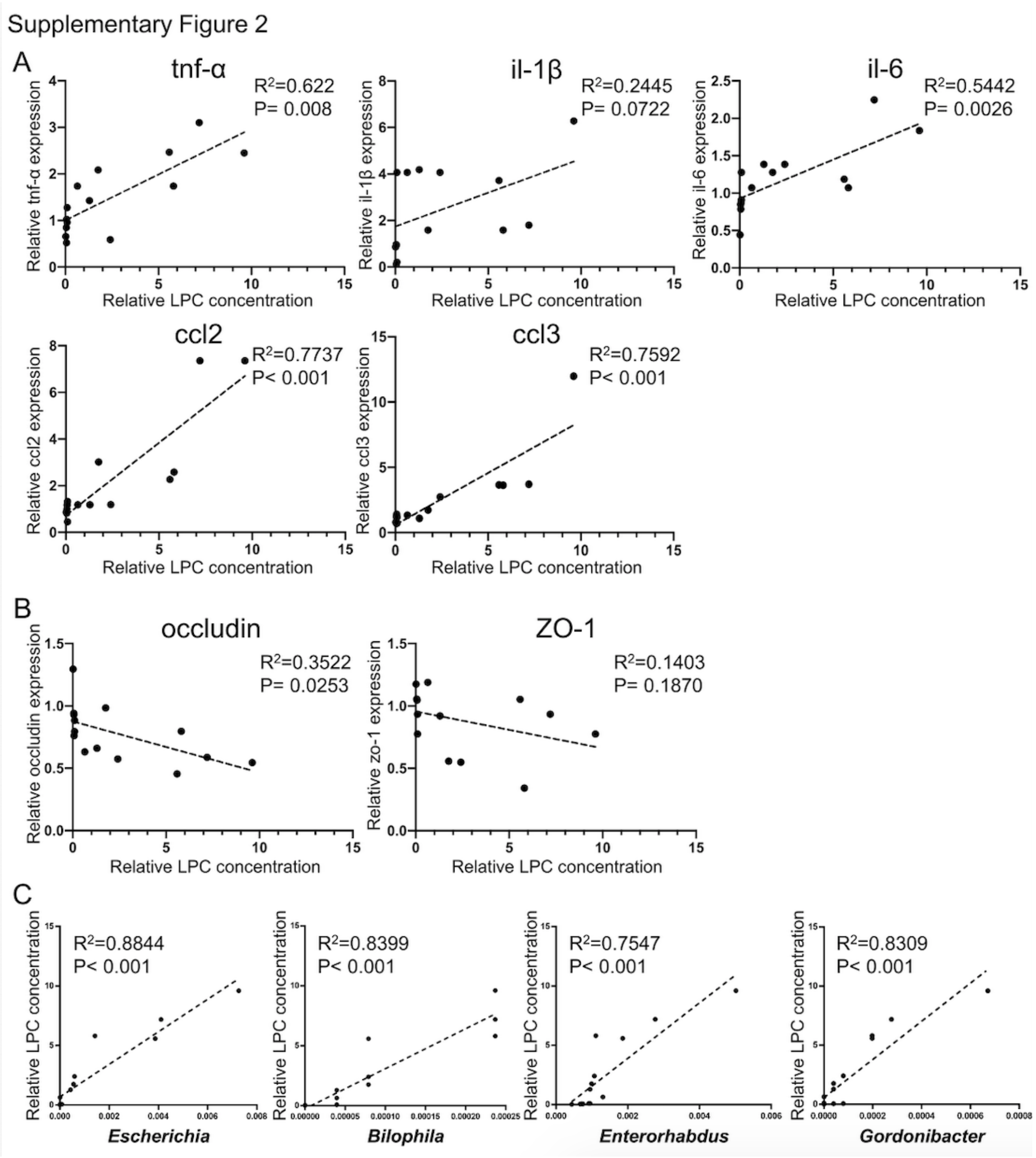

Supplementary Figure 2. LPC concentration was positively correlated with colonic pro-inflammatory cytokines and negatively correlated with tight junction genes. It was also correlated with gut microbiota. A) Correlation analysis of LPC and pro-inflammatory cytokines, *tnf-α* (P=0.008) , *il-1β* (P=0.0722), *il6* (P=0.0026), *ccl2* (P＜0.001), *ccl3* (P＜0.001). (B) Correlation analysis of LPC and epithelial tight junction genes—*occludin* (P=0.0253) and ZO-*1* (0.1870). (C) LPC was positively correlated with *Escherichia*, *Bilophila*, *Enterorhabdus* and *Gordonibacter*. (n=14)
